# Nurses' Registration

**Published:** 1891-01-03

**Authors:** 


### NURSES' REGISTRATION.

A different, but not less worthy, class of persons have also sought for State registration. That class consists of a section, a minority, of trained nurses. That minority sought to gain the approval of the General Medical Council in the early part of the year, and of the British Medical Association in the autumn. In neither case was it successful. The General Medical Council did not think it was the proper body to take in hand the registration of trained nurses, and the British Medical Association declined to discuss the question during the present year. A body more intimately connected with nurses and nursing than either of the two named took still more decisive steps in the matter. This organisation consisted of representatives of almost all the nurse training schools both in London and the country. It prepared a memorandum setting forth fundamental objections to the State registration of trained nurses, and took measures to render its objections effectual. It seems to be the fact that the wisest matrons, nurses, medical men, and hospital managers, cannot see that State registration would confer any solid advantages upon nurses, whilst it might and would impose certain undoubted disadvantages. The scheme would cost money, which the nurses alone would have to pay; and it would restrict liberty without providing compensating protection. There seems a probability that unless it can be shown that definite and practical advantages to the nurses will certainly result from State registration, the demand for it will gradually die out.

